# Policy silences: why Canada needs a National First Nations, Inuit and Métis health policy

**DOI:** 10.3402/ijch.v72i0.22690

**Published:** 2013-12-27

**Authors:** Josée G. Lavoie

**Affiliations:** School of Health Sciences, University of Northern British Columbia, Prince George, British Columbia, Canada

**Keywords:** indigenous populations, health services, indigenous, health policy, legislation, Canada

## Abstract

**Objectives:**

Despite attempts, policy silences continue to create barriers to addressing the healthcare needs of First Nations, Inuit and Métis. The purpose of this article is to answer the question, *if what we have in Canada is an Aboriginal health policy patchwork that fails to address inequities, then what would a Healthy Aboriginal Health Policy framework look like?*

**Methods:**

The data collected included federal, provincial and territorial health policies and legislation that contain Aboriginal, First Nation, Inuit and/or Métis-specific provisions available on the internet. Key websites included the Parliamentary Library, federal, provincial and territorial health and Aboriginal websites, as well as the Department of Justice Canada, Statistics Canada and the Aboriginal Canada Portal.

**Results:**

The Indian Act gives the Governor in Council the authority to make health regulations. The First Nations and Inuit Health Branch (FNIHB) of Health Canada historically provided health services to First Nations and Inuit, as a matter of policy. FNIHB's policies are few, and apply only to Status Indians and Inuit. Health legislation in 2 territories and 4 provinces contain no provision to clarify their responsibilities. In provinces where provisions exist, they broadly focus on jurisdiction. Few Aboriginal-specific policies and policy frameworks exist. Generally, these apply to some Aboriginal peoples and exclude others.

**Conclusion:**

Although some Aboriginal-specific provisions exist in some legislation, and some policies are in place, significant gaps and jurisdictional ambiguities remain. This policy patchwork perpetuates confusion. A national First Nation, Inuit and Métis policy framework is needed to address this issue.

The Canadian health system consists of inter-related components that are the responsibility of the federal, territorial, provincial, municipal governments, First Nation authorities, or the private sector (1). What glues the system together is legislation, policies, relationships and goodwill. In some cases, this results in a relatively seamless system. In most cases however, the system is at best loosely woven, resulting in gaps and ambiguities (2).

The fragmented nature of the healthcare system, to which jurisdictional issues add complexity and confusion, creates a patchwork of policies and programmes for First Nations, Inuit and Métis (hereafter Aboriginal peoples when discussed as a group, (3). In some cases, goodwill-based initiatives and relationships mitigate policy shortfalls, and facilitate access. While helpful, vesting access to essential health services in goodwill is a concern, and has been shown to be insufficient: evidence suggests that access to healthcare services continues to be problematic (4, 5). When services are accessed, responsiveness is not assured (6).

This article draws on data collected for a project undertaken for the National Collaborating Centre for Aboriginal Health (NCCAH), with funding from the Public Health Agency of Canada (PHAC). The NCCAH is 1 of 6 national collaborating centres established by the PHAC to renew and strengthen public health in Canada. The overall mandate of that study was broader than what can be reported here. The final report is available at www.nccah.ca

For the purpose of this broader project, we undertook a review of Aboriginal-specific provisions entrenched in national, territorial or provincial legislation and policy documents, a review of Treaties and self-government agreements, and explored Aboriginal organizations’ mandates. This article focuses only on a subset of this data: the Aboriginal-specific provisions entrenched in health policies and legislation. This article examines what is and is not articulated in these documents, and documents policy silences, to answer the question, *if what we have in Canada is an Aboriginal health policy patchwork that fails to address inequities, then what would a Healthy Aboriginal Health Policy framework look like?* This article begins with a brief discussion of the methodology. This is followed by a section that explores federal, territorial and provincial legislation and policies, highlights strengths and gaps. A final section discusses the need for the adoption of a National First Nations, Inuit and Métis Health Policy, based on common principles that nevertheless reflect First Nations, Inuit and Métis diversity.

## Methodology

This article draws on a dataset developed based on a comprehensive review of Aboriginal-specific provisions embedded in health policies and legislation available on the internet. Policies and legislation are formal documents that shape institutional arrangements. Although legislation can be amended or repealed and policies replaced, their formality denotes a longer term commitment. Of course, legislation and policies tell only part of the story: informal arrangements can also create opportunities for bridging jurisdictional gaps. However, informal arrangements are not documented and can change quickly at the whim of changes in government, cut-backs, and changes in staff. Informal arrangements are also difficult to track down.

Table I summarizes the type of documents that were analyzed to inform this article, and the retrieval strategies used. The documents were compiled over an initial 1-year period (March 2007–April 2008), and updated to 2013 for this article. Federal, provincial and territorial health legislation, policies and strategies implemented from 1970 onward were included. Key websites explored included: the Parliamentary Library; Health Canada; the PHAC; Aboriginal Affairs and Northern Development Canada (AANDC, then Indian and Northern Affairs Canada, INAC); Department of Justice Canada; Statistics Canada; the Aboriginal Canada Portal; provincial and territorial websites including Ministries/Departments of Aboriginal Affairs and Ministries/Departments of Health.

**Table I:** Type of document retrieved and methodology.

Category	Type of document reviewed and focus
Treaties and self-government activities	• Historical treaties; • Modern treaties, also known as land claim agreements; • Self-government agreements; and • Agreements specific to health, such as those that emerged as a result of the Health Transfer Policy. Although not all documents speak directly to health issues, each document was scanned for the words “health,” “medicine,” “medical” and “doctor.” We included 1 or 2 sentences to briefly describe if and how health is referenced in these documents. For those documents where health may not be specifically mentioned, we included them in this report if they are important in the context of Aboriginal health in Canada.
Federal legislation, policies and Aboriginal health	• The Department of Canadian Heritage, which houses an Aboriginal Affairs Branch, and is responsible for 33 Acts. • The Federal Healthcare Partnership (FHP), which was established in 1994. • Citizenship and Immigration Canada which provides health services to certain classes of migrants (primarily refugee claimants and Convention refugees) in need of assistance during their settlement period in Canada. • Correctional Service Canada, which provides health services to federal inmates and some former inmates on parole. • The Department of National Defence, which provides health services to regular Force members and eligible members of the Reserve Force. • Health Canada, which is responsible for health services to eligible First Nations peoples and Inuit, through the First Nations and Inuit Health Branch. • The Royal Canadian Mounted Police which provides services to its regular members, eligible civilian members (i.e. civilian members injured during the course of their duties), and eligible retired members (i.e. retired members in receipt of a disability pension where the disability is work-related); and • Veterans Affairs Canada, which provides health services to eligible veterans and others who qualify for its programmes. • The Public Works and Government Services Canada, the Treasury Board of Canada Secretariat and the Public Health Agency of Canada participate in FHP discussions. • The Department of Indian and Northern Affairs Canada has the federal responsibility for Indian Affairs. Indian health was initially included under Indian Affairs.
Provincial/territorial legislation, policies and Aboriginal health	**Existing territorial and provincial legislation and policies**: Information at the provincial and territorial levels is not uniformly available. A number of provinces and territories list legislation and regulations by department or ministry. In that case, the number of legislation (Acts) for which each ministry or department is responsible for was recorded. However, if they were not listed by department or ministry, then the government legislation website was word searched and each located document was word searched. All successful word searches were recorded. Key words included Aboriginal, First Nation, *Amérindiens*, Inuit, Métis or Metis, Indians, health, doctor, medicine and medical. **Decentralization – Regionalization of Health Services:** The purpose of decentralizing healthcare systems to regional health authorities is in part to increase public participation in decision making. Bands and Tribal Councils have been one of the few means of engagement available to First Nations, especially in remote and rural areas. We reviewed legislation and policies to identify provisions for First Nation, Inuit, Métis and Aboriginal participation.

Internet searches included the following terms and combinations of these words: Aboriginal, First Nation(s), Inuit, Metis or Métis, Indian, Amérindiens, Reserve, Health and Medical. Lower case was used to avoid problems retrieving data from case-sensitive search engines. The data presented are based on information that is publicly available on the internet. This method was chosen for a number of reasons. First, the decision was made to limit this project to publicly and readily available information to ensure consistency. Second, the internet is an important tool of policy research and information for policy makers, researchers, users and many government departments. Third, expanding this project to include documents that are not readily available on the internet would have required identifying key collaborators within each government department and training them to ensure consistency in information gathering. This would have required considerably more resources and time, to possibly yield little more than what was available on the internet. A key limitation of this project is that the internet is a challenging research tool. The information is forever shifting and no consistent method for referencing has been uniformly adopted. Accuracy is at times difficult to ascertain, and must be checked against numerous sources. Furthermore, there is no way to ascertain that the record is complete. Therefore, the data reported here may have gaps in information.

## Findings

The starting point of any discussion on jurisdiction is the Constitution Act 1867, which established that Indians were a federal jurisdiction. In the 1939 decision, the Supreme Court of Canada settled the issue and determined that the Inuit were “Indians” under the British North America Act, 1867 and thus also a federal responsibility. The federal government provides a complement of health services provided to registered Indians living on-reserve and to Inuit living in their traditional territories in Québec and Labrador. Only one programme applies to all registered Indians and to Inuit, regardless of where they live: the Non-Insured Health Benefits (NIHB) programme.

Provincial and territorial governments are responsible for the delivery of a number of health services, as defined by the Canada Health Act 1984, complemented by services designed to meet territorial or provincial priorities. Métis, off-reserve registered Indians, non-registered Indians, and Inuit living outside of their traditional territories fall under the purview of territorial and provincial governments. As the level of services delivered in different provinces/territories may vary, that means that the level of services provided to Aboriginal peoples as residents across provinces and territories will vary.

While theoretically relatively clear, a number of intersecting federal, provincial and territorial legislation, policies and authorities with shifting and blurred responsibilities contribute to ambiguities and gaps, as discussed below.

### Federal jurisdiction

The federal government's obligation over Indian health is spelled out in Section 73 of the Indian Act, which gives the Governor in Council the authority to make certain regulations related to medical treatment, and public health (7). The Indian Act does not outline an obligation to provide services, and does not provide sufficient authority for a comprehensive public health and health service regulatory framework on First Nations reserves. To date, no court challenge has focused on Section 73, and therefore no jurisprudence exists to further clarify the extent of federal obligations.

There are only 2 national health policies relevant to this study: the 1979 Indian Health Policy and the 1989 Health Transfer Policy. The Indian Health Policy was adopted on 19 September 1979 (8). The policy was a 2-page document with one broad-based objective:

the goal of Federal Indian Health Policy is to achieve an increasing level of health in Indian communities, generated and maintained by the Indian communities themselves. (9)

There is ambiguity as to the range of application of the Indian Health Policy, because the text of the policy does not specify whether it is inclusive of registered and non-registered Indians. It makes no mention of Inuit.

The Health Transfer Policy is the most tangible outcome of the Indian Health Policy. It was rolled out by the First Nations and Inuit Health Branch of Health Canada (FNIHB) in 1989, and provided opportunities for single communities and Tribal Councils to assume the responsibility for the planning and delivery of community-based health services, as well as some regionally based programmes (10). The objective of the policy was to promote community uptake of community-based health services, as well as some regional programmes provided by FNIHB. The Health Transfer Policy applies to First Nations on-reserve and to the Inuit of Labrador only.

The PHAC was created in 2004 by an Act of Parliament (11). PHAC currently offers a number of off-reserve health programmes specifically designed to meet the needs of marginalized populations, including Aboriginal peoples living off-reserve. In its 2007–2012 Strategic Plan (which is still the most recent plan to date), PHAC stated the need to increase its capacity in Aboriginal health and to develop a strong overarching strategic Aboriginal public health policy. To do this, PHAC proposed to launch and maintain collaborative relationships with national and regional Aboriginal organizations and other federal departments (12). Provincial and territorial jurisdictions were not mentioned.

As can be seen from the discussion above, the federal policy framework informing issues of jurisdiction over Aboriginal health is thin and loosely woven. The framework is silent on the Métis and on those who are not eligible for registration as Indians under the Indian Act. The policy framework does not link to a legislative framework, other than the Indian Act and the Canada Health Act 1984. Provisions under these Acts are broadly worded.

### Provincial and territorial jurisdiction

Findings for territorial and provincial health legislative frameworks are summarized in Table II. As shown, no specific provision exists in the Northwest Territories, Nunavut, British Columbia, Manitoba, Nova Scotia and Prince Edward Island health legislation to clarify these territories and provinces’ responsibilities in Aboriginal health. Provisions that exist fall into 3 broad categories. The first group includes provisions stating that the minister may opt to enter into an agreement with Canada and/or First Nations for the delivery of health services (Alberta, Saskatchewan, Ontario and New Brunswick), thereby clearly indicating that services on-reserve are outside of the province's mandate. The second group includes provisions clarifying the application of legislation on Métis settlements (Alberta only).

**Table II:** Legislative and policy patchwork: the territories and provinces.

	YK	NWT	NU	BC	AB	SK	MB	ON	QC	NB	NS	PEI	NFLD & LAB
Provisions stating that the minister may enter into an agreement with Canada and/or First Nations for the delivery of health services.					√	√		√		√			
Provision clarifying the application of legislation on Métis settlements.					√								
Provisions related to existing modern treaties.	√								√				√
Territory/province-wide Aboriginal health framework.		√		√				√			√		
Health legislation recognizing the need to respect traditional healing practices.	√												
Provisions that recognize that Aboriginal traditional healers should be exempted from control specified under the Code of Professions.								√					
Provisions in tobacco control legislation stating that the legislation does not apply to the use of tobacco for ceremonial purposes.						√	√	√		√		√	

The final group relates to health legislation in the Yukon, Quebec, and Newfoundland and Labrador, that contain provisions clarifying these territory/provinces’ roles and responsibilities in health in areas included in self-government. For example, while the Yukon Health Act stipulates the importance of partnerships with Aboriginal groups and the respect of traditional Aboriginal healing, it also stipulates that the Yukon Land Claim Agreement or the Yukon First Nations Self-Government Agreement shall prevail in a conflict (13). Similar provisions exist in Quebec, and Newfoundland and Labrador.

Some legislation includes provisions related to traditional practices. The Yukon is the only jurisdiction where health legislation recognizes the need to respect traditional healing practices, and the importance of establishing partnerships with Aboriginal peoples (13). Ontario recognizes that Aboriginal midwives and traditional healers should be exempt from control specified under the Code of Professions. Specific provisions are listed under the Midwifery Act (14). Finally, Saskatchewan, Manitoba, Ontario, New Brunswick and Prince Edward Island have adopted tobacco control legislation that specifies that the legislation does not apply to the use of tobacco for ceremonial purposes (15–19).

Findings also show the existence of a limited number of Aboriginal-specific policies/frameworks. Ontario was the first province to develop an Aboriginal Health and Wellness Strategy in 1990 and to develop an overarching Aboriginal Health Policy in 1994 (20). The Aboriginal Health Policy is intended to act as a governing policy and to assist the Ministry of Health in accessing inequities in First Nation/Aboriginal health programming, responding to Aboriginal priorities, adjusting existing programmes to respond more effectively to needs, supporting the reallocations of resources to Aboriginal initiatives, and improving interaction and collaboration between ministry branches to support holistic approaches to health. This is the most comprehensive policy framework currently in place in Canada. The Northwest Territories is the only jurisdiction with a Métis Health Policy (21). However, the policy is limited to extending access to NIHB as provided to Registered Indians.

In British Columbia, the 2005 *Transformative Change Accord and the First Nations Health Plan* form a Tripartite First Nations policy framework that aims to close the disparities that exist between First Nations and other British Columbians in the areas of health, education and housing (22, 23). The framework also intends to clarify issues of Aboriginal titles and jurisdiction. The framework explicitly applies to First Nations, and does not address the needs of other Aboriginal groups in British Columbia (24, 25). A similar framework was developed in Nova Scotia. The 2005 *Providing Health Care, Achieving Health – Mi'kmaq* focuses on the specific needs of the Mi'kmaq people; however, it does not address the needs of the Métis and other Aboriginal peoples living in Nova Scotia (26).

### Decentralization

Most provinces (with the exception of Prince Edward Island and more recently Alberta) and the Northwest Territories have adopted decentralized models of healthcare delivery. Decentralization is a transfer of authority from the Department of Health to regional health authorities (RHAs) tasked with priority setting and the allocation and management of health resources (27). Decentralization is intended to increase opportunities for citizen engagement in local priority setting.

Our findings show that most decentralized provincial healthcare systems have not entrenched mechanisms to ensure Aboriginal representation on RHAs. British Columbia (28) and Nova Scotia (29) have provisions that stipulate that the make-up of the Board of Directors must reflect the population that the RHAs are set up to serve. Aboriginal peoples are not specifically mentioned. Ontario is the only province to have established a council composed of Aboriginal peoples to advise on regional priority setting in healthcare, which is provided through the Local Health Integration Networks (30).

### Self-government activities

A variety of arrangements have emerged in the past 3 decades, adding further complexity to jurisdictional issues. In some areas, the numbered Treaties signed between 1870 and 1929 remain the most current expression of self-government activities. In others, self-government agreements have been signed that clarify areas of ambiguities embedded in historical treaties (for example, Ref. (31). Self-government agreements have also been signed in areas where historical treaties had never been negotiated (for example, the 1974 James Bay and Northern Quebec Agreement; the 1993 Nunavut Lands Claim Agreement, and the 1999 Agreement with the Nisga'a Nation).

Some self-government agreements have established Aboriginal government's jurisdiction in health. The Nisga'a Agreement, the James Bay and Northern Quebec Agreement, and the Labrador Inuit Association Agreement are tripartite agreements that include clarification of jurisdiction over health services, roles and responsibilities, as well as mechanisms to address jurisdictional issues as they emerge. Still, each agreement is somewhat unique, thereby creating somewhat different arrangements and obligations. To date, Newfoundland and Labrador, the Yukon and Quebec have responded by embedding provisions in their legislation to clarify issues of jurisdiction resulting from these agreements. Some provinces, Alberta, Saskatchewan, Ontario and New Brunswick, have adopted provisions stating that the minister may opt to enter into an agreement with Canada and/or First Nations for the delivery of health services, thus providing a mechanism for clarifying issues of jurisdiction in communities where self-government agreements have been signed. Other provinces have remained silent on this matter.

## Discussion

The data explored above constitute the Aboriginal health legislative and policy framework that exists in the provinces and the territories. It shows that what exists is very much a jurisdictional patchwork. Legislative frameworks show little evidence of concern for addressing Aboriginal needs: the main focus remains the clarification of jurisdiction, and even that is partial. Policy frameworks are few. While progress has been made, there is considerable variation from one province/territory to the next and significant gaps.

When taken together, federal and provincial/territorial legislative and policy frameworks fail the test of seamlessness. They also fail to address shifts in jurisdiction related to changes in legislation, decentralization (or recentralization), self-government activities, or as a result of other arrangements. Areas that are particularly problematic are highlighted below.

*Sub-populations poorly served* by the current frameworks include First Nation individuals who are recognized as a member of a First Nations through Band rules but are nevertheless not eligible for registration under the Indian Act. Funding for health services is, however, calculated on the basis of the population actually served only in communities where services are provided by Nursing Stations (16% of First Nations communities). In all other communities, FNIHB funds communities for services delivered to registered Indians only (10). The number of children and adults who are not eligible for registration under the Indian Act as a result of Bill C-31 and Bill C-3, and who nevertheless live on-reserve, is growing (32, 33). In terms of health services, these individuals exist in jurisdictional limbo. First Nations organizations must decide to provide services for all, at a loss, or to provide services only to those members for whom they receive funding, while remaining politically accountable to all members and thereby risking political fallouts.

There is a *growing number of individuals of First Nations ancestry* who live off-reserve and who do not qualify for registration under the Indian Act. Although the responsibility for providing care to these individuals falls under the purview of the provinces and territories, the responsiveness of provincial services has been questioned (34–37). With the exception of Ontario, and emerging dialogues in Nova Scotia and British Columbia, current policies and legislation have yet to entrench provisions to improve the responsiveness of provincial health services.

Recently, cross-jurisdictional mechanisms have emerged in a few provinces. Examples include:

- the Saskatchewan Northern Health Strategy (ended in 2010) that brought together First Nations, Métis, northern municipalities, RHAs, and federal and provincial authorities; and
- the Inter-Governmental Committee on Manitoba First Nations Health whose membership includes representatives from the Assembly of Manitoba Chiefs, the Manitoba Keewatinook Ininew Okimowin,the Southern Chiefs Organization Inc., FNIHB – Manitoba Region, the PHAC, Manitoba Health, the Manitoba Department of Aboriginal and Northern Affairs, Family Services and Housing Manitoba, Manitoba Finance, and AANDC.

Whilst encouraging, these mechanisms are not empowered to change legislation and adopt policies, and their effectiveness in addressing cross-jurisdictional issues is constrained by existing legislation, policies and budgets that are decided at the national and provincial levels.

The analysis provided above suggests that although some areas of jurisdiction are clear or clearer, shifts related to changes in the Indian Act, new Aboriginal self-government and tripartite agreements to improve access to health services, have added and will continue to add complexities. This suggests that Aboriginal health jurisdictional boundaries will continue to shift and blur over time. A national mechanism is required, and this mechanism does not exist.

## Conclusion

Canada needs an overarching national mechanism, a National First Nations, Inuit and Métis Health Policy, to realize improvements in Aboriginal health through federal, provincial and territorial healthcare systems. This proposal comes, of course, with 2 broad challenges. First, the federal–provincial jurisdictional divide is often believed to preclude the adoption of nationwide approaches that nevertheless are expected to influence provincial and territorial governments in an area that is defined in the Constitution Act as a provincial jurisdiction. While the concern is legitimate, the example of the Canada Health Act 1984 illustrates that a national act can effectively guide provincial and territorial healthcare systems through voluntary membership, shared principles and financial incentives. The Canada Health Act may serve as a model, where federal departments, territories and provinces may voluntarily sign on in their commitment to close jurisdictional gaps and health inequalities. Principles to be imbedded into this policy framework would be defined in negotiation with First Nations, Inuit and Métis organizations.

The second challenge may come from First Nations, Inuit and Métis themselves who have rejected pan-Aboriginal approaches en bloc, and who may object of the adoption of a national Aboriginal approach simply because it is likely to gloss over key differences, contexts and priorities. The concern is valid and important; however, it may not be a significant obstacle if engagement occurs at the onset, and if the output – the policy – provides opportunities for First Nations, Inuit and Métis to pursue their priorities, based on their values and aspirations. The experience of the past 40 years should have taught us that critical and systematic engagement is the only mechanism that will yield a credible product, and it is the only way forward.
